# A commentary on ‘Local resection for solid pseudopapillary neoplasms of the pancreas shows improved postoperative gastrointestinal function and reduced mental stress: a multiquestionnaire survey from a large cohort’

**DOI:** 10.1097/JS9.0000000000001651

**Published:** 2024-05-20

**Authors:** Tingtong Lin, Xiaoqi Zhang, Jiehao Xiao, Dandan Li

**Affiliations:** a The Second Clinical Medical College, Lanzhou University; b The Second Department of General Surgery, The First Hospital of Lanzhou University, Lanzhou, Gansu Province, People’s Republic of China

*Dear Editor,*

Solid pseudopancreatic tumor (SPN) is a rare, low-grade malignant pancreatic tumor with an incidence of ~2–3% of all pancreatic tumors, which has risen in recent years probably due to advancements in medical knowledge and imaging diagnostic skills related to this disease^1^. The main population of SPN is young women, with an average onset age of 28, coinciding with a crucial stage of personal and professional advancements^1^. Consequently, the onset of this illness can significantly impact the lives and careers of those affected. Currently, the preferred treatment for SPN is surgical resection, whether to choose radical resection (RR) or local resection (LR) depends on various factors such as tumor location, resection difficulty, and local infiltration^1^. Lymph node dissection needs to be considered if lymph node metastasis or local infiltration occurs which however rarely happens^2^. While studies indicate that the majority of patients opt for RR to minimize the risk of recurrence, LR offers the advantage of preserving pancreatic function, theoretically improving gastrointestinal function (GIF), and reducing mental stress. However, there is a lack of concrete research evidence to support these claims^3^. We greatly appreciate the groundbreaking work by Gao and colleagues, as demonstrated in their study titled ‘Local resection for solid pseudopapillary neoplasms of the pancreas shows improved postoperative gastrointestinal function and reduced mental stress: a multiquestionnaire survey from a large cohort.’ This study confirms that LR and minimally invasive surgery (MIS) can improve GIF and reduce mental stress, offering valuable guidance for surgeons in devising personalized treatment plans. However, several issues warrant further consideration and discussion.

First, this study is a single-center investigation that lacks diversity, with a limited sample size of only 183 valid questionnaires collected. Second, being a cross-sectional study, it is difficult to determine a cause-and-effect relationship but only suggest an association between variables. Third, the validity of the questionnaire remains to be considered. It only mentions criteria such as completing all questions, answering quality control questions correctly, and spending more than 4 min on the survey, without specifying the quality control criteria. Moreover, the online survey consists of eight questionnaires, usually leading respondents to submit incomplete or arbitrarily answered surveys if deemed excessively long^4^. Additionally, the inclusion of patients diagnosed between October 2001 and April 2021 introduces potential recall bias, particularly among earlier patients. Fourth, the authors employed multiple linear regression models to explore the relationship between patients’ GIF, mental stress and various factors but failed to consider the potential interaction effects of these factors, as the 183 patients included 46 LR and 137 RR, 89 open surgery and 94 MIS, different treatment options for different patients, such as adopting MIS while performing LR surgery, might yield better results, but it is difficult to determine exactly which combination played a central role for the patients. Lastly, the presence of potential covariates, including personal and extrinsic factors, necessitates further exploration. Patient baseline information, encompassing medical history, including but not limited to gastrointestinal and mental disorders, and easily overlooked mental states, requires detailed elucidation. For instance, the inclusion of the PHQ-9 (Patient Health Questionnaire-9) depression scale in the questionnaire could introduce bias if patients initially suffered from depression. Moreover, personality traits prone to anxiety may exacerbate fear and worry about the illness, affecting GIF and emotional stress^5^. Therefore, it’s crucial to acknowledge that differences in GIF and mental stress among patients undergoing RR versus LR may not solely stem from the surgical procedures themselves but could also be influenced by various factors as mentioned. Additionally, external factors such as patients’ work environment, dietary habits, life stress and other aspects of the factors should not be ignored. Moreover, given that patients were included during the COVID-19 pandemic from 2020 to 2021, some adverse effects on patients’ emotions and postoperative prognosis may need to be taken into account^6^.

Overall, we offer much appreciation to Gao and his team for studying the impact of LR on patients’ postoperative GIF and mental stress, offering valuable insights for surgeons in formulating more precise and personalized treatment plans. However, further research is needed to bolster the credibility of the findings presented in the paper.

## Ethical approval

Not applicable.

## Consent

Not applicable.

## Sources of funding

This research received no particular grant from any funding agency in the public, private, or not-for profit sectors.

## Author contribution

All authors read and approved the final version of the manuscript.

## Conflicts of interest disclosure

The authors declare no conflicts of interest.

## Research registration unique identifying number (UIN)

1. Name of the registry: not applicable.
2. Unique identifying number or registration ID: not applicable.
3. Hyperlink to your specific registration (must be publicly accessible and will be checked): not applicable.

## Guarantor

Tingtong Lin.

## Data availability statement

No data was generated during the writing of this study.

## Provenance and peer review

Not applicable.
